# Gambling on visual performance: neural correlates of metacognitive choice between visual lotteries

**DOI:** 10.3389/fnins.2015.00314

**Published:** 2015-09-03

**Authors:** Shih-Wei Wu, Mauricio R. Delgado, Laurence T. Maloney

**Affiliations:** ^1^Institute of Neuroscience, National Yang-Ming UniversityTaipei, Taiwan; ^2^Brain Research Center, National Yang-Ming UniversityTaipei, Taiwan; ^3^Department of Psychology, Rutgers UniversityNewark, NJ, USA; ^4^Department of Psychology, New York UniversityNew York, NY, USA; ^5^Center for Neural Science, New York UniversityNew York, NY, USA

**Keywords:** decision under risk, metacognition, second-order judgment, valuation, medial prefrontal cortex, striatum, intraparietal sulcus, lateral prefrontal cortex

## Abstract

A lottery is a list of mutually exclusive outcomes together with their associated probabilities of occurrence. Decision making is often modeled as choices between lotteries and—in typical research on decision under risk—the probabilities are given to the subject explicitly in numerical form. In this study, we examined lottery decision task where the probabilities of receiving various rewards are contingent on the subjects' own visual performance in a random-dot-motion (RDM) discrimination task, a metacognitive or second order judgment. While there is a large literature concerning the RDM task and there is also a large literature on decision under risk, little is known about metacognitive decisions when the source of uncertainty is visual. Using fMRI with humans, we found distinct fronto-striatal and fronto-parietal networks representing subjects' estimates of his or her performance, reward value, and the expected value (EV) of the lotteries. The fronto-striatal network includes the dorsomedial prefrontal cortex and the ventral striatum, involved in reward processing and value-based decision-making. The fronto-parietal network includes the intraparietal sulcus and the ventrolateral prefrontal cortex, which was shown to be involved in the accumulation of sensory evidence during visual decision making and in metacognitive judgments on visual performance. These results demonstrate that—while valuation of performance-based lotteries involves a common fronto-striatal valuation network—an additional network unique to the estimation of task-related performance is recruited for the integration of probability and reward information when probability is inferred from visual performance.

## Introduction

Decision making is often modeled as a choice among lotteries: each lottery is a list of mutually exclusive outcomes paired with their probabilities of occurrence. A decision maker might choose between a lottery (0.5, $500;0.5, $0) representing a 50% chance of receiving $500 and a 50% chance of receiving nothing, and (0.95, $250;0.05, $0) a 95% chance of $250 and a 5% chance of receiving nothing. A central focus of research on decision making has been to understand how people use and integrate information about probability and outcome in making decisions (Kahneman and Tversky, 1979). Behavioral research indicated that human choices often deviate from the predictions of standard economic model (Kahneman and Tversky, 2002). For example, there is accumulating evidence suggesting that people tend to distort probability information. Much effort has been dedicated to modeling such distortion (e.g., Tversky and Kahneman, 1992; Gonzalez and Wu, 1999).

Most neurobiological studies to date have focused on decision under risk (Knight, 1921) with both probability and value information given to the subject in explicit form (e.g., Hsu et al., 2009) or probability learned from sampling experience (e.g., Fiorillo et al., 2003). The networks recruited are typically involved in reward processing and valuation (e.g., see Kable and Glimcher, 2009; Bartra et al., 2013, for review). Midbrain dopaminergic system and the associated fronto-striatal networks in particular have also been associated with coding reward probability (Fiorillo et al., 2003; Volz et al., 2003; Knutson et al., 2005; Preuschoff et al., 2006; Hsu et al., 2009; FitzGerald et al., 2010; Wu et al., 2011; for review, see Platt and Huettel, 2008). Reward probability is represented by phasic response, while risk (variance) is represented by tonic response in the midbrain dopaminergic neurons and in the ventral striatum (Fiorillo et al., 2003; Preuschoff et al., 2006). The ventral striatum and the medial prefrontal cortex have been shown to represent subjective value of lottery options in both decision under risk and decision under ambiguity (Levy et al., 2010), suggesting that these regions are involved in the integration of probability and outcome information in decision tasks with different levels of uncertainty.

But there are many sources of uncertainty that we can gamble on. We can gamble on the weather or the traffic to the airport and often do so. We can also gamble on our own visual and motor abilities, a form of metacognition (Fleming et al., 2012b) or second-order judgment (Barthelmé and Mamassian, 2009).

An evident question is, given that there are many possible sources of probability, to what extent do the neural substrates of decision making overlap for tasks differing in source of probability? One possibility—the common-network hypothesis—is that probability information from all sources converges to a common representation and only then is this probability information combined with value information to reach a decision. Under this hypothesis, any decision is computed in the same valuation networks known to represent probability information in decision tasks with explicitly described lotteries or lotteries learned from sampling experience (e.g., Platt and Huettel, 2008; Bartra et al., 2013). Our first goal is to test this common-network hypothesis.

We constructed lotteries where probabilities of reward are specified as the chances of success in a well-known visual discrimination task—the random-dot-motion (RDM) discrimination task. On each trial the subject saw two lotteries with binary outcomes each based on a different visual RDM stimulus and with different monetary rewards assigned. In this visual lottery decision task, the subject was instructed to choose the lottery she or he preferred with the understanding that, at the end of the experiment two of the chosen lotteries would be randomly selected and realized. To realize a lottery, the subject performed a one-shot RDM discrimination at it. He or she won the associated reward for making a correct judgment on dot motion direction and otherwise nothing.

The unique feature of the visual lottery decision task is how information about reward probability is obtained. For each lottery under consideration, it is required that subjects estimated the probability of making a correct visual judgment in order to infer reward probability. It is plausible that this task-related estimation recruits additional networks during valuation of the visual lotteries. But it is unknown whether the networks recruited are those identified in previous research concerning the RDM task as a visual discrimination task.

Convergent evidence points to the involvement of the posterior parietal cortex (PPC) and the lateral prefrontal cortex (LPFC) in accumulating sensory evidence over time to guide visual discrimination of dot motion direction (Roitman and Shadlen, 2002; Heekeren et al., 2006; for reviews, see Gold and Shadlen, 2007; Heekeren et al., 2008). This line of work highlights how the brain arrives at a judgment during the RDM task. However, unlike performance in the RDM task, what is needed in our task is an estimate of the probability that the first-order judgment is correct, a second-order judgment. Interestingly, research on metacognition has shown these regions represent second-order judgment of visual and memory performance (Fleming et al., 2012a; Baird et al., 2013). The rostrolateral prefrontal cortex has been shown to be more active when subjects are engaged in metacognitive judgment of performance in a perceptual decision-making task compared with a control task (Fleming et al., 2012a). Activity in this region correlates with subjects' confidence in their first-order judgment (Hebart et al., 2014). For memory-related metacognitive judgment, resting-state fMRI data reveals that the connectivity between the intraparietal sulcus and the medial prefrontal cortex correlates with individual difference in behavioral metacognitive performance (Baird et al., 2013).

Our second hypothesis—the second-order representation hypothesis—is the claim that the metacognitive probability of success in carrying out the RDM task is computed in the PPC-LPFC network, the same network shown to be involved in both the first-order and second-order judgments of visual performance. The common-network hypothesis and the second-order representation hypothesis are logically independent of each other: one may be false without implications for the other. In this study, we tested them both.

## Materials and methods

### Constructing a visual lottery decision task

In decision-making under risk, subjects in laboratory experiments are typically presented with pairs of lotteries and asked to choose the one they prefer in each pair. If the probabilities and reward values are explicitly given, the choice between lotteries is a form of decision under risk (Knight, 1921).

To design a visual task that is mathematically equivalent to a typical economic lottery, we modified the random-dot motion (RDM) discrimination task, a well-established paradigm for studying visual decision-making. In the standard RDM task, a visual stimulus consisting of a patch of moving dots is presented to subjects. A proportion of dots moved coherently in one of two possible directions (in our case, up or down), while the remaining dots moved at random in all possible directions with equal probability. The subject's task was to indicate the direction of coherent dot motion. Correct responses were rewarded.

**Figure 1:**
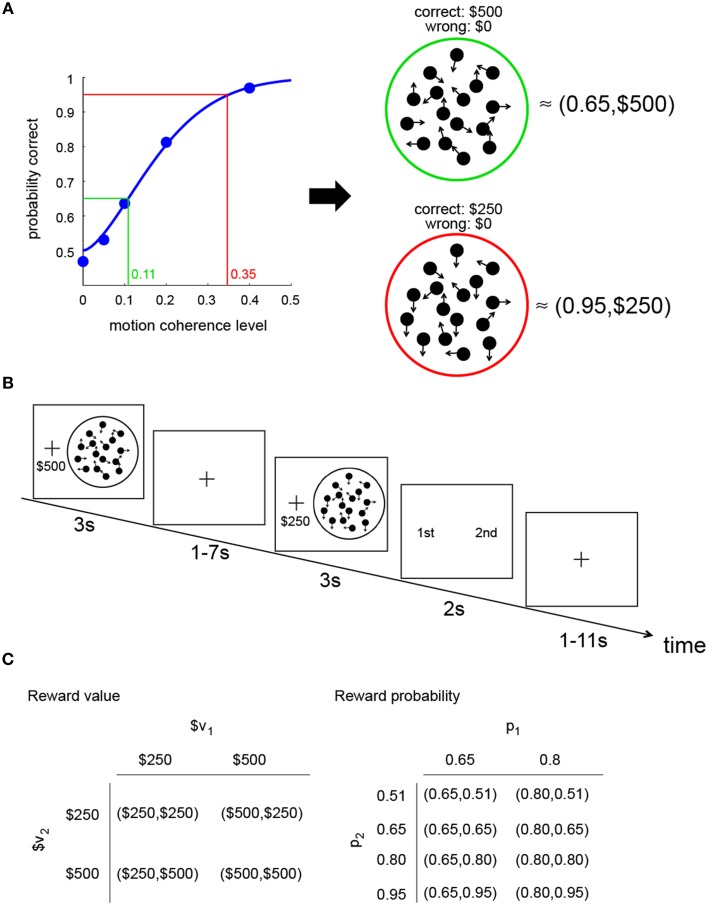
**Constructing a visual lottery decision task**. **(A)** Construction of two visual lotteries. The psychometric function (blue curve) describes the relation between the probability of making a correct judgment on dot motion direction and motion coherence level in the RDM task. Based on the psychometric function estimated for each subject separately, we can construct pairs of “visual lotteries.” Each lottery corresponds to a RDM stimulus. In each lottery, we associate correct response with a monetary reward and incorrect response with no reward. In this example, a lottery (0.65,$500) is equivalent to a RDM stimulus that had 0.11 coherence, while a lottery (0.95,$250) is equivalent to a RDM stimulus with 0.35 coherence. **(B)** Trial sequence of the visual lottery task. **(C)** Design: combinations of reward value and probability in the visual lottery decision task. The values in each lottery pair were assigned at random, independent of each other, as were the probabilities. The rewards and probabilities of each lottery were also assigned independently.

The relation between performance (probability of correct response) and motion coherence level (the proportion of dots moving coherently in one direction) is illustrated in Figure 1A. As the coherence level increases, the probability of making a correct response increases. The blue curve in the graph represents the psychometric function estimated from the performance of an actual subject in this experiment.

Since the subjects receive a reward for making a correct response, the probability of a correct response is the probability of reward (*p*). Performance at chance level is 50% correct; the range of *p* is therefore between 0.5 and 1. We manipulated *p* within this range by changing the coherence level. Based on the psychometric function unique to the subject, for any probability of reward ranging from 0.5 to asymptotic level of performance, we could find the motion coherence level that matches it. We also manipulated the reward value $*x* given for a correct response.

In the example shown in Figure 1A, the RDM stimulus with green ring has a coherence level of 0.11 and was equivalent to a lottery (0.65,$500;0.35,$0) while the RDM stimulus with red ring has a coherence level of 0.35 and is equivalent to a lottery (0.95,$250;0.05,$0). We referred to each such pairing of an RDM stimulus with a reward value as a perceptual lottery. The RDM stimuli shown here are purely for illustration purposes; they do not reflect what the subjects actually saw in the experiment.

To reliably estimate the psychometric function for each subject, we first trained the subject on the standard RDM task (see Section Session 1 in Procedure). Following the training session, the subjects came back on the next day to do the visual lottery decision task. For more details, see Section Session 2 in Procedure.

#### Procedure

There were two sessions in the experiment, both carried out in the MRI scanner. The fMRI results reported in this paper represent the results from Session 2: the visual lottery decision task. The two sessions fell within 2 days.

##### Session 1: Random-dot-motion discrimination task

The goals of this session were to train the subjects to perform the standard RDM discrimination task in the MRI scanner and to estimate the psychometric function for the subsequent visual lottery decision session (Session 2) that was the focus of this experiment.

In this session, the subjects were engaged in a fixed-duration version of the RDM task. Each trial started with a fixation cross at the center of the screen (0.5 s). A small reward value was selected at random from [1, 2, 4, 8] NTD (1 US$ = 30 National Taiwan Dollar) and presented beneath the fixation cross. The subjects were instructed that they would receive the reward if they made a correct judgment on dot motion direction. The RDM stimulus then appeared for a fixed duration of 3 s. The subjects were instructed to fixate the cross and use only his/her peripheral vision to evaluate the RDM stimulus.

The RDM stimulus was followed and replaced by a response screen. The subjects had up to 2 s to make a button-press response to indicate his/her decision on motion direction. A variable inter-stimulus interval (ISI) (1–7 s uniform distribution in steps of 1 s) followed after the response and before feedback was provided. At feedback, the subjects were notified whether his/her judgment was correct. The feedback was presented for 0.5 s and was followed by a variable inter-trial interval (ITI; 1–11 s uniform distribution in steps of 1 s) before the next trial.

There were five runs in the session. Each run consisted of 40 trials. The subjects encountered five possible coherence levels in each run (0, 0.05, 0.1, 0.2, and 0.4; eight trials per coherence level). Each coherence level occurred on eight trials in a run and 40 trials in total across five runs resulting in a total of 200 trials to estimate the psychometric function.

##### Session 2: Visual lottery decision task

The decision task was a choice between two visual lotteries. On each trial, the subjects were successively presented with two visual lotteries and asked to choose between them. The example in Figure 1B illustrates the trial sequence. In this example, the first perceptual lottery presented (L1) is equivalent to (0.65,$500;0.35,$0), while the second perceptual lottery (L2) is equivalent to (0.95,$250;0.05,$0). After seeing both lotteries, a choice screen would appear and the subjects would select the preferred lottery (L1 or L2) by pressing a button.

After viewing each visual lottery, subjects were not required to indicate his or her judgment on the direction of dot motion. She or he had only to indicate which lottery she or he preferred by pressing one of two buttons. The subjects were told that—at the end of the experiment—two of the lotteries she or he chose during the session would be selected at random. For each selected lottery the subject would execute the RDM task, judging the direction of motion. If he or she judged successfully, he or she received the reward value associated with the lottery. The sum of the winnings from the two lottery attempts was paid to the subject as a bonus. To maximize his or her expected winnings, for each lottery under consideration, the subject had to estimate his or her own probability of making a correct judgment of dot motion—a second-order judgment—and integrate it with the reward value associated with a correct performance.

Both the reward value and reward probability associated with the first lottery (L1) were varied independently of those for the second lottery (L2). In Figure 1C, we illustrate the lottery design. Let L1: (p_1_,$v_1_) and L2: (p_2_,$v_2_). As shown in the figure, there were four combinations of $v_1_ and $v_2_. Each combination had eight trials in a run. Since there were two levels of p_1_ (0.65 and 0.8) and four levels of p_2_ (0.51, 0.65, 0.8, 0.95), we had eight combinations of p_1_ and p_2_. Each combination was repeated four times in a run. The four possible [$v_1_,$v_2_] and the eight possible [p_1_,p_2_] combinations were randomly assigned to the trials. As a consequence, there were 32 distinct trials in a run. There were a total of five runs in the session, for a total of 160 trials.

Following the presentation of L2, the choice screen appeared and subjects had up to 2 s to indicate his/her choice (L1 or L2). The location of L1 and L2 was randomized across trials. After making a response, feedback indicating which lottery was chosen (L1 or L2) appeared for 0.5 s and was followed by an ITI (1–11 s drawn from a uniform distribution in steps of 1 s) before the next trial.

#### Subjects

Twenty-five subjects participated in the experiment. After session 1, each subject's performance was analyzed. If a subject's performance, i.e., percentage of correct response, did not increase monotonically as a function of motion coherence level, we did not invite the subject back to the second session. Among the 25 subjects, six subjects participated only in the first session and did not continue on to the second. Thus, a total of 19 subjects completed the experiment (10 women, mean age = 24). All subjects gave written informed consent prior to participation in accordance with the procedures approved by Taipei Veteran General Hospital IRB. Their average earning was 1923 NTD (1 US$ = 30 National Taiwan Dollar or NTD).

#### Stimuli

The task was programmed using the Psychophysics Toolbox in MATLAB (Brainard, 1997; Pelli, 1997). The scripts for the RDM stimuli were obtained from https://www.shadlenlab.columbia.edu and subsequently modified for the current study. The stimuli were presented to the subjects through MRI-compatible goggles (Resonance Technology Inc.). Because the stimuli were presented through these goggles, we were not able to provide stimulus-related measures in terms of visual angle. We thus described them in pixel size. The screen resolution was set to 800 × 600 pixels. The frame rate was 60 Hz. The aperture was 112 pixels in diameter. At stimulus onset, seven dots were presented within the aperture. At each subsequent frame, a subset of dots moved coherently in one direction while the others moved randomly. Once a dot moved out of the aperture, a new dot was drawn at the opposite side of the aperture. Within each frame, only seven dots were shown.

#### fMRI data acquisition

Imaging data were collected with a 3T MRI scanner (Siemens Tim Trio) equipped with a 12-channel head array coil. T2^*^-weighted functional images were collected using an EPI sequence (TR = 2 s, TE = 30 ms, 33 oblique slices acquired in ascending interleaved order, 3.4 × 3.4 × 3.4 mm isotropic voxels, 64 × 64 matrix in a 220-mm field of view, flip angle 90°). Each run consisted of 304 images. T1-weighted anatomical images were collected using an MPRAGE sequence (TR = 2.53 s, TE = 3.03 ms, flip angle = 7°, 192 sagittal slices, 1 × 1 × 1 mm isotropic voxel, 224 × 256 matrix in a 256-mm field of view).

#### fMRI preprocessing

The following pre-processing steps were applied using FMRIB's Software Library (FSL) (Smith et al., 2004). First, motion correction was applied using MCFLIRT to remove the effect of head motion during each run. Second, we applied spatial smoothing using a Gaussian kernel of FWHM 8 mm. Third, a high-pass temporal filtering was applied using Gaussian-weighted least square straight line fitting with σ = 50 s. Fourth, registration was performed using a two-step procedure. First, the unsmoothed EPI image that was the midpoint of the scan was used to estimate the transformation matrix (seven-parameter affine transformations) from EPI images to the subject's high-resolution T1-weighted structural image, with non-brain structures removed using FSL's BET (Brain Extraction Tool). Second, we estimated the transformation matrix (12-parameter affine transformation) from the high-resolution T1-weighted structural image with non-brain structures removed to the standard MNI template brain.

### General linear models of bold response

To analyze fMRI data from Session 2 (visual lottery decision task), we estimated four general linear models (GLM) of the BOLD responses and one psychophysiological interaction (PPI) model using FSL's FEAT module (fMRI Expert Analysis Tool). The first two GLMs were used for the whole-brain analyses (GLM-1 and GLM-2), while GLM-3, GLM-4 and the PPI model were conducted for region-of-interest (ROI) analyses. As described in Section Procedure, each trial consisted of two phases, lottery presentation and choice response (a button-press response to indicate lottery choice). We focused the GLM analyses on the lottery presentation phase. The GLMs described below list the regressors implemented for the modeling of this phase. They differ only in this phase.

#### GLM-1

(R1) Indicator function for the duration of lottery presentation (3 s), (R2) R1 multiplied by the reward probability (*p*) of the currently presented lottery, (R3) R1 multiplied by the reward value (*$v*) of the currently presented lottery. The contrast of [R1 R2 R3] = [0 1 0] was used to examine the neural correlates of reward probability of the visual lottery. The contrast of [R1 R2 R3] = [0 0 1] was used to test the neural correlates of reward value of the visual lottery.

#### GLM-2

(R1) Indicator function for the duration of lottery presentation (3 s), (R2) R1 multiplied by the EV of the currently presented lottery. The contrast of [R1 R2] = [0 1] was used to examine the neural correlates of EV of the visual lottery.

#### GLM-3

(R1) Indicator function for the duration of lottery presentation of L1 (3 s) when the correct motion direction was upward, (R2) R1 multiplied by the EV of L1 (EV_L1_), (R3) Indicator function for the duration of lottery presentation of L1 (3 s) when the correct motion direction was downward, (R4) R3 multiplied by EV_L1_, (R5) Indicator function for the duration of lottery presentation of L2 (3 s) when the correct motion direction was upward, (R6) R5 multiplied by the EV of L2 (EV_L2_), (R7) Indicator function for the duration of lottery presentation of L2 (3 s) when the correct motion direction was downward, (R8) R7 multiplied by EV_L2_. The contrast of [R1 R2 R3 R4 R5 R6 R7 R8] = [0 1 0 0 0 1 0 0] was used to test the neural correlates of EV of the visual lottery when the correct motion was upward, [0 0 0 1 0 0 0 1] was used to test the neural correlates of EV of the visual lottery when the correct motion was downward, [0 1 0 1 0 0 0 0] for EV of L1, and [0 0 0 0 0 1 0 1] for EV of L2.

#### GLM-4

(R1) Indicator function for the duration of each lottery presentation (3 s) when L1 was chosen, (R2) R1 multiplied by the EV of the currently presented lottery, (R3) Indicator function for the duration of each lottery presentation (3 s) when L2 was chosen, (R4) R3 multiplied by the EV of the currently presented lottery. The contrast of [R1 R2 R3 R4] = [0 1 0 0] was used to test the neural correlates of EV of the visual lotteries on trials when the subject chose L1, and [0 0 0 1] for EV of the visual lotteries on trials when the subjects chose L2.

For the choice phase, we implemented the following three regressors in all the above GLMs: An indicator function, a parametric regressor for EV_C_–EV_NC_ (the difference in EV between the Chosen and Non-Chosen lotteries) and a parametric regressor for EV_L2_–EV_L1_ (the difference in EV between L2 and L1). For each trial, the duration of these regressors was the subject's reaction time. All the parametric regressors in the GLMs were *z*-normalized so as to allow for comparisons of their beta estimates when necessary. Each regressor in a GLM was convolved with a canonical gamma hemodynamic response function. We implemented temporal derivatives of each regressor in each model as the regressors of no interest. This implementation often serves as an alternative to slice-timing correction and is useful to model for non-linear neural and vascular effects on the timing shift of BOLD response (Calhoun et al., 2004).

All GLM analyses were carried out in the following steps (Beckmann et al., 2003). First, BOLD time series were pre-whitened with local autocorrelation correction. A first-level FEAT analysis was carried out for each run of each subject. Second, a second-level fixed-effect (FE) analysis was carried out for each subject that combined the first-level FEAT results from different runs using the summary statistics approach. Finally, a third-level mixed-effect (ME) analysis using FSL's FLAME module (FMRIB's Local Analysis of Mixed Effects) was carried out across subjects by taking the FE results from the previous level and treating subjects as a random effect (Woolrich et al., 2004). All reported whole-brain results were corrected for multiple comparisons. We first identified clusters of activation by defining a cluster-forming threshold of the z statistic. Then, a family-wise error corrected *p*-value of each cluster based on its size was estimated using Gaussian random field theory (Worsley et al., 1992).

### Region-of-interest (ROI) analyses

The identification of independent, unbiased ROIs was based on two methods, which we describe below.

#### ROI method I

Following Esterman et al. (2010) and Litt et al. (2010), we created an independent, unbiased ROI using the leave-one-subject-out approach. That is, we created an ROI in a particular region for each subject separately by the following steps. First, for each subject, we performed a mixed-effect analysis on the contrast of interest using all other subjects' data. As a result, we obtained a statistical parametric map (SPM) of that contrast. The SPM obtained for each subject was thus independent of that subject's data. We then performed the standard cluster-based statistical inference to correct for multiple comparisons using Gaussian random field theory (cluster-forming threshold *z* = 2.5, family-wise error-corrected *p* < 0.05). The cluster(s) that was significant were then used as the independent mask(s).

#### ROI method II

A key question in this study was to investigate the role of sensory systems involved in processing visual motion in the visual lottery decision task (Session 2). This required us to perform ROI analysis on area V5/MT+. Since we did not use a functional task to identify area V5/MT+, we used the anatomical mask based on an existing atlas. Here we selected the Jülich histological atlas, which was created by averaging multi-subject post-mortem cyto- and myelo-architectonic segmentations (Eickhoff et al., 2005) and had been shown to have strong overlap with functionally identified V5/MT+ (Wilms et al., 2005). We used this atlas to create the left V5/MT+ROI and the right V5/MT+ROI.

### Psycho-physiologic interaction (PPI) analyses

Area V5/MT+ has been shown to represent the coherence level of the visual motion information and critically involved in forming a judgment of motion direction in the RDM discrimination task (Gold and Shadlen, 2007). As such, it served as an important seed region for our connectivity analysis. Specifically, we conducted a PPI analysis (Friston et al., 1997) motivated by the following question: how might the functional connectivity of V5/MT+ with the regions that represent the expected value (EV) of the visual lotteries change during the course of a trial in the visual lottery decision task (Session 2)?

To address this question, we used the left and right anatomically identified V5/MT+ROIs as seed regions. For each seed, we extracted and deconvolved its mean BOLD time series data (Gitelman et al., 2003). The GLM had the following regressors: (R1) The deconvolved and de-meaned seed time course (often referred to as the physiological regressor), (R2) Indicator function for the duration of each lottery presentation (3 s), (R3) R2 multiplied by the EV of the currently presented lottery, (R4) Indicator function for the duration of the delay between L1 and L2 (1–7 s), (R5) R4 multiplied by EV_L1_, (R6) An indicator function for the choice period (duration = reaction time), (R7) R6 multiplied by EV_C_–EV_NC_, (R8) R6 multiplied by EV_L2_–EV_L1_, (R9) Interaction between R1 and R2, (R10) Interaction between R1 and R4, (R11) Interaction between R1 and R6. The PPI contrasts labeled as “lottery,” “delay,” and “choice” in **Figures 6B,C** correspond to R9, R10, and R11, respectively. All regressors were convolved with a canonical gamma hemodynamic response function. Temporal derivatives of each regressor were included in the model as regressors of no interest.

## Results

### Behavioral results: Random-dot-motion discrimination task

**Figure 2:**
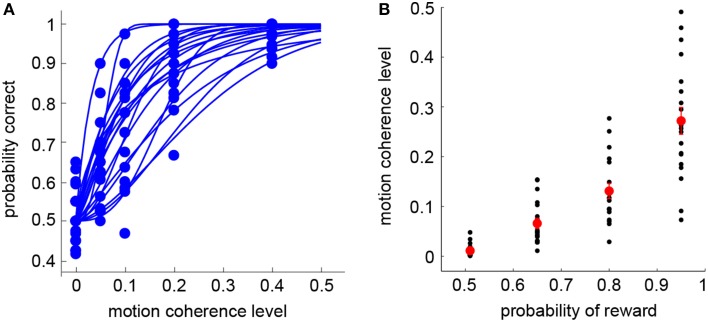
**Behavioral results: RDM task (Session 1)**. **(A)** Psychometric functions in the RDM discrimination task (Session 1). Each curve represents a single subject. **(B)** Motion coherence levels used in the visual lottery decision task (Session 2) are plotted against their corresponding probabilities of reward. The black dots indicate data from individual subjects. The red dots indicate the mean of the motion coherence level averaged across subjects. Error bars represent ±1 SEM.

Subjects first completed the standard RDM discrimination task (Session 1). For each subject, we separately estimated the psychometric function that described the relation between the probability of making a correct judgment on dot motion direction and the motion coherence level (Britten et al., 1992). Overall, subjects' performance was at chance level (50% correct) at zero coherence, and reached 90% correct or above when coherence was 0.4 (see Figure 2A—each curve represents the psychometric function of a single subject). Once the psychometric function was obtained, the visual lottery pairs were tailored for each subject separately in the subsequent visual lottery decision task (Session 2). In Figure 2B, we plot the motion coherence level (y-axis) that corresponds to the probabilities of reward used in the visual lottery task (x-axis). Note that the probability of reward is equivalent to the probability of making a correct judgment. There were four possible reward probabilities, [0.51 0.65 0.8 0.95] (see Section Materials and Methods). For each subject, the motion coherence level corresponding to a particular reward probability was calculated based on his/her psychometric function. The black dots represent individual subjects' data, while the red dots were the mean coherence level averaged across subjects.

### Behavioral results: Visual lottery decision task

**Figure 3:**
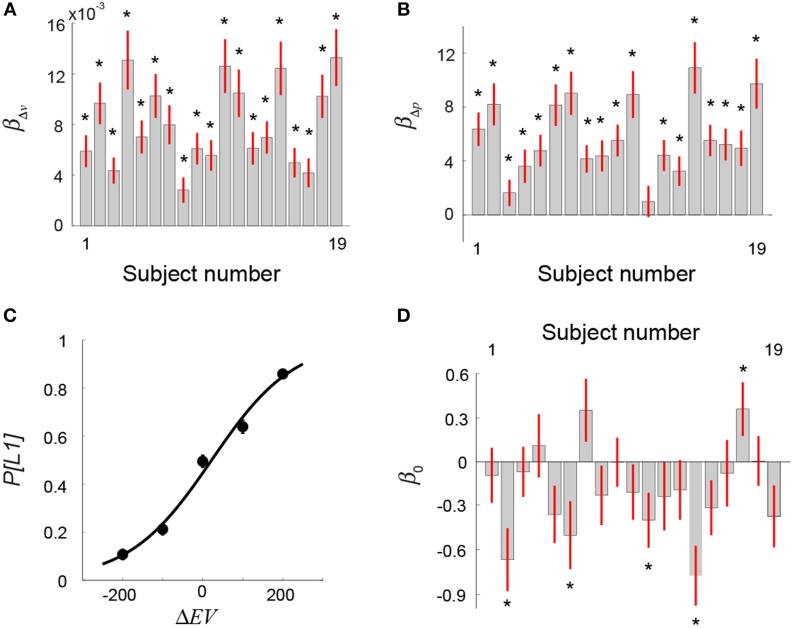
**Behavioral results: Visual lottery decision task (Session 2). (A,B)** Logistic regression analysis on choice against difference in reward value (Δ*v*) and on reward probability (Δ*p*) between the two lotteries. **(A)** Beta estimates of Δ*v* separately estimated for each subject. **(B)** Beta estimates of Δ*p* separately estimated for each subject. **(C)** Mean frequency of choosing the first lottery (*P[L1]*) (averaged across subjects) as a function of the difference in *EV* between L1 and L2 (Δ*EV* = *EV*_*L*1_−*EV*_*L*2_). **(D)** Beta coefficient of the intercept (β_0_) in the logistic regression analysis on choice that used Δ*EV* as the regressor (Equation 1 in Results). The star symbol indicates statistical significance (*p* < 0.05, one-sample *t*-test). Error bars represent ±1 SEM.

For each subject, we performed a logistic regression analysis by regressing choice behavior against the difference in reward probability (Δ*p*) between the two lotteries and the difference in reward value (Δ*v*). If a subject took into account both the probability and reward information when making decisions, the beta estimates for Δ*p* (β_Δ*p*_) and Δ*v* (β_Δ*v*_) would both be significantly different from zero. Figures 3A,B show the results. We found that all but one subject's β_Δ*p*_ was significantly >0 (one-sample *t*-test on each subject, *p*-value thresholded at 0.05) (Figure 3B), while all subjects' β_Δ*v*_ were significantly >0 (*p* < 0.05, one-sample *t*-test on each subject) (Figure 3A). This indicated that subjects made use of both probability information and reward values in selecting lotteries. We also ran a model with the interaction of Δ*p* and Δ*v* added in addition to Δ*p* and Δ*v*. The beta estimates of Δ*p* and Δ*v* were similar to the model that did not include the interaction term. The statistical conclusion of β_Δ*p*_ and β_Δ*v*_ did not change with the interaction added to the model. On the other hand, we found that the beta estimate of the interaction term was not significant in 18 out of 19 subjects (*p* < 0.05, one-sample *t*-test on each subject).

We next examined how EV influenced choice. For a lottery (p,$v), the *EV* = *p*·*v*. We estimated a psychometric choice function describing the relation between the probability of choosing L1 and the difference between the EV of the first and second lotteries based on the following equation

(1) $$P\left[L_{1}\right]=\frac{1}{1+\exp\left[-\left(\beta_{0}+\beta_{1}\Delta EV\right)\right]}$$

where *P*[*L*1] represents the probability of choosing L1 and Δ*EV* = *EV*_*L*1_−*EV*_*L*2_. Estimating this psychometric choice function allows us to explicitly test whether subjects exhibited significant bias toward L1 or L2 as a result of the sequential presentations. In Figure 3C, we plot the mean choice probabilities averaged across subjects as a function of Δ*EV* and the psychometric function. We found that the indifference point (the estimate of β_0_) did not differ significantly from Δ*EV* = 0 (one-sample *t*-test, *t* = −0.2, *df* = 18, *p* = 0.84), indicating that subjects on average did not show a bias toward either L1 or L2. For each subject separately, we also estimated his or her psychometric choice function. The results of individual subjects' estimate of β_0_ are plotted in Figure 3D. The beta estimate of 14 out of 19 subjects did not differ significantly from zero (one-sample *t*-test on each subject, *p* < 0.05), indicating no bias toward either L1 or L2. Among the five subjects that showed a significant bias, β_0_ of four subjects was significantly smaller than zero, indicating a bias toward L2.

### Neural correlates of valuation in the visual lottery decision task

In the visual lottery decision task, the two lotteries in each trial were presented sequentially. When a lottery was presented, information about reward value and the probability of obtaining it were revealed. As described earlier, in order to estimate the probability of receiving the reward associated with each lottery under consideration, subjects needed to estimate his or her own probability of making a correct judgment based on the noisy motion stimulus presented. This is a form of metacognition or second-order judgment.

**Figure 4:**
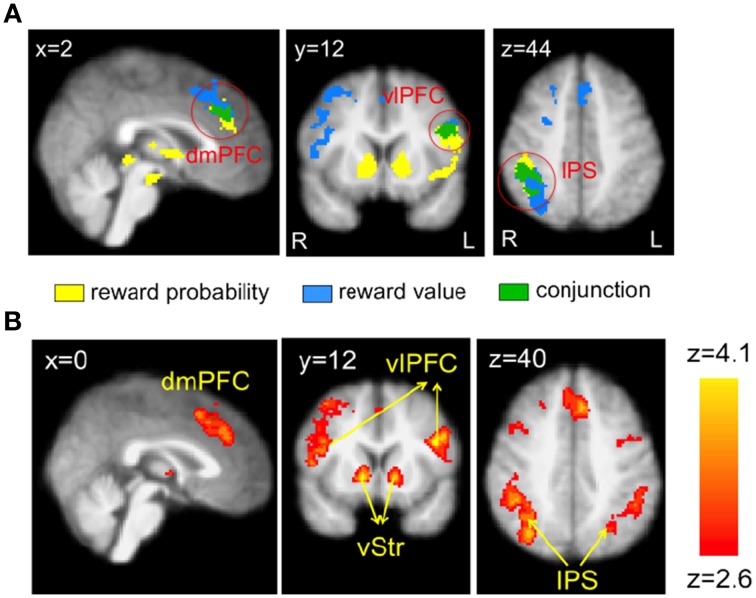
**Neural correlates of reward probability, reward value, and expected value (EV) of the visual lottery**. **(A)** Neural correlates of reward probability (yellow), reward value (blue), and their conjunction (green). **(B)** Neural correlates of EV. Regions in the dorsomedial prefrontal cortex (dmPFC), bilateral ventrolateral prefrontal cortex (vlPFC), ventral striatum (vStr), and bilateral intraparietal sulcus (IPS) correlated with the expected value of the visual lotteries in the decision task when lottery information was revealed. Statistical inference was based on family-wise error-corrected *p*-values (*p* < 0.05) on cluster size (cluster-forming threshold *z* = 2.6) using Gaussian random field theory.

Table 1 summarizes the regions that correlated with reward probability (GLM-1), reward value (GLM-1), and the EV (GLM-2) of the visual lottery. BOLD response in lateral prefrontal, dorsomedial prefrontal, and posterior parietal cortices correlated with both reward probability and reward value (Figure 4A). Statistical images were thresholded at *z* = 2.6 and binarized for display purposes. Additionally, we found six clusters that correlated with EV: the dorsomedial prefrontal cortex (dmPFC), the ventral striatum (vStr), the left ventrolateral prefrontal cortex (vlPFC), the right vlPFC, the left intraparietal sulcus (IPS), and the right IPS (Figure 4B). We referred to these six regions as the EV-coding regions.

**Table 1:** **Regions in which the BOLD signal was positively correlated with reward probability, reward value, and the expected value (EV) of the visual lottery**.

**Brain region**	**Hemisphere**	**Voxels**	***z*-Max**	***x***	***y***	***z***
**REWARD PROBABILITY**						
Lateral orbitofrontal cortex/anterior insula	L	1970	4.3	−36	30	−6
Ventral striatum	R	1499	4.4	12	12	−2
Intraparietal sulcus	R	887	3.5	44	−34	46
Lateral orbitofrontal cortex/anterior insula	R	734	4.2	32	22	−8
Dorsomedial prefrontal cortex	L	488	3.8	−8	36	34
**REWARD VALUE**						
Intraparietal sulcus	R	1359	3.7	44	−46	46
Ventrolateral prefrontal cortex	R	894	3.6	52	20	28
Ventrolateral prefrontal cortex	L	628	3.6	−44	10	26
Dorsomedial prefrontal cortex	L	596	3.7	−8	28	38
Dorsolateral prefrontal cortex	R	497	3.7	24	18	54
**EV**						
Ventrolateral prefrontal cortex	R	1958	3.7	42	40	14
Intraparietal sulcus	R	1949	4.1	42	−46	46
Ventral striatum	R	1810	4.0	12	12	−2
Ventrolateral prefrontal cortex	L	1406	4.2	−44	10	26
Dorsomedial prefrontal cortex	L	1018	3.9	−8	28	38
Intraparietal sulcus	L	781	3.5	−28	−64	34

*Clusters that survived cluster-based correction (z > 2.6, family-wise error-corrected p < 0.05 using Gaussian random field theory). The z-max column represented the MNI coordinates of the maximum z-statistic*.

The EV signals found in these regions represent the EV associated with an option/good, i.e., the visual lottery. By design, the EV of a lottery is independent of its motion direction. Hence, the EV signals found here should not reflect the visual aspects of the task such as the direction of dot motion. Further, subjects in this task were asked to choose between two lotteries rather than making a decision about motion direction. This is different from the task the subjects perform in the standard RDM task in which they are explicitly asked to indicate judgment of dot motion direction. Hence, the EV signals found here should not reflect the value associated with a motor action to indicate motion direction. Finally, the lottery-button mapping was not revealed during the lottery presentations. Hence, the subjects did not know what button to press to indicate his or her lottery choice when the lotteries were presented. The EV signals therefore cannot be tied to a particular motor action implementing subjects' choice.

### ROI analysis on the robustness of EV-coding

To further examine the robustness of EV representations in the EV-coding regions described above (dmPFC, vStr, left vlPFC, right vlPFC, left IPS, right IPS), we performed four additional ROI analyses on each one of them. The ROIs were identified using ROI method I (see Section Materials and Methods). The first analysis tested whether these regions represented both reward probability and reward value (GLM-1). The second analysis tested whether these regions represented EV independent of sensory attribute of the visual lotteries, i.e., the direction of coherent dot motion (GLM-3). The third analysis tested whether these regions represented EV independent of the choices the subjects made (GLM-4). The fourth analysis tested whether these regions represented EV of both L1 and L2 (GLM-3).

**Figure 5:**
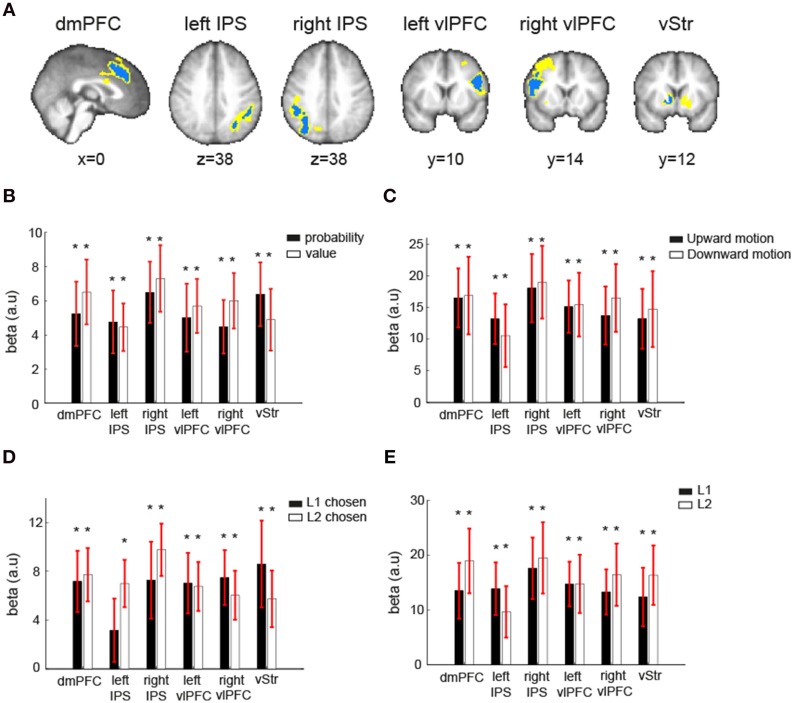
**Robustness of EV-coding. (A)** Independent ROIs in six regions that correlated with expected value (EV) of the visual lottery under consideration were created using the leave-one-subject-out approach. Because each ROI was created separately for each subject, the ROIs across subjects in one region did not completely overlap. The blue indicated the common voxels shared across all subjects' ROIs, while the yellow indicated the voxels that were from at least one subject's ROI. **(B)** Mean beta estimates of the reward probability (black) and reward value (white). **(C)** Mean beta estimates of expected value (EV) of the visual lottery at its presentation when the correct motion direction was upward (black) or downward (white). **(D)** Mean beta estimates of EV of the visual lottery at its presentation when the first lottery presented in a trial was eventually chosen (L1 chosen, black) or when the second lottery was chosen (L2 chosen, white). **(E)** Mean beta estimates of EV of the first visual lottery presented in a trial (L1, black) and the second lottery presented in a trial (L2, white). The star symbol indicates statistical significance (*p* < 0.05, one-sample *t*-test for each contrast at each ROI). Error bars represent ±1 SEM.

The results are shown in Figure 5. For each contrast of interest shown in the figure, the beta estimates are the mean beta estimates averaged across subjects. Each mean beta estimate at each ROI was tested against zero using one-sample *t*-test with *p*-value at 0.05. First, these regions represented both reward probability and reward value, as their beta estimates were both significantly different from zero at each ROI (Figure 5B). Second, the beta estimates of EV when the motion direction was upward and when it was downward were both significantly different from zero (Figure 5C). Third, the beta estimates of EV were significantly different from zero when subjects chose L1 and when they chose L2 (Figure 5D). The only exception is the left IPS, which did not significantly correlate with EV of the lotteries when L1 was chosen. Fourth, the beta estimates of EV for L1 and for L2 were both significantly different from zero (Figure 5E).

### Dynamic interaction analysis between the EV-coding regions and V5/MT

**Figure 6:**
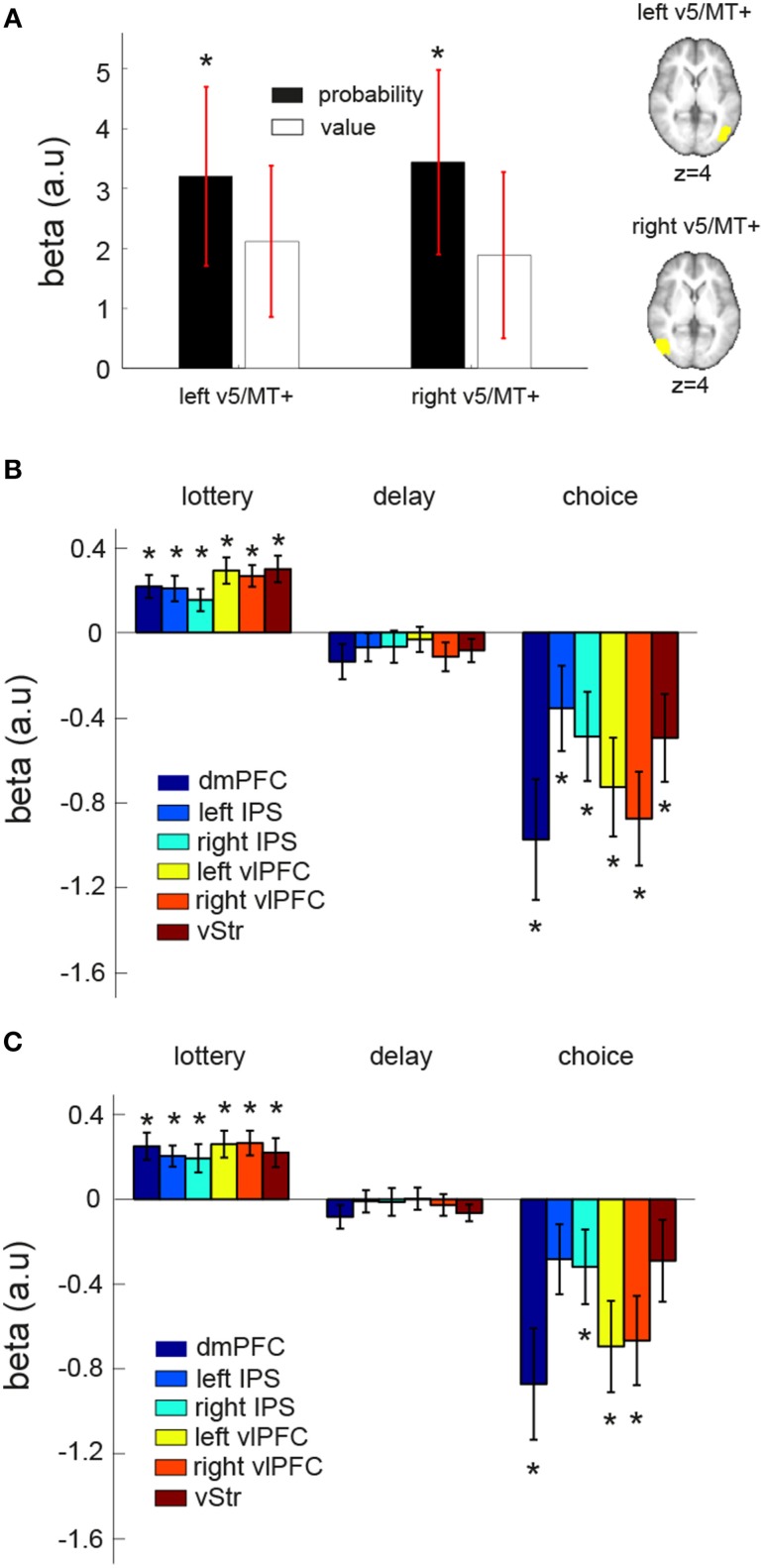
**ROI and connectivity analyses on area V5/MT+**. **(A)** Anatomically defined left and right V5/MT+ showed significant correlation with reward probability (black) but not reward value (white) at the time when lotteries were presented. (**B,C)** Psycho-physiologic interaction (PPI) analysis. We investigated the changes in functional connectivity between a seed region (left V5/MT+ or right V5/MT+) and the EV-coding regions (dmPFC, vStr, left vlPFC, right vlPFC, left IPS, right IPS) at different stages during a trial: “lottery” indicates the time where the lottery information was revealed, “delay” indicates the fixation period between the first lottery and the second lottery, and “choice” indicates the time where subjects made a button-press response to indicate his or her lottery choice. The mean beta estimate of each PPI contrast of each EV-coding region is plotted. **(B)** Left V5/MT+ as the seed region. **(C)** Right V5/MT+ as the seed region. The star symbol indicates statistical significance (*p* < 0.05, one-sample *t*-test for each contrast at each ROI). Error bars represent ±1 SEM.

Convergent evidence suggests that area V5/MT+ is involved in analyzing visual motion. V5/MT+ activity has been shown to positively correlate with motion coherence level in the standard RDM task (Gold and Shadlen, 2007) and to predict behavioral performance (Newsome et al., 1989; Salzman et al., 1990). In our whole-brain results, we did not find BOLD response to correlate with EV in area V5/MT+ after correction for multiple comparisons. We subsequently performed two analyses on anatomically defined V5/MT+ (see Section ROI method II in Materials and Methods for details). The first analysis revealed that V5/MT+ significantly correlated with reward probability, but not reward value. In Figure 6A, mean beta estimate (across subjects) of reward probability (black) and reward value were plotted separately for the left and right V5/MT+.

In the second analysis, we performed a psycho-physiologic interaction (PPI) analysis to examine the dynamic interaction between V5/MT+ and the six identified EV-coding regions during the course of a trial. The results are summarized in Figures 6B,C. The value of the beta estimates (averaged across subjects for each contrast at each ROI) shown in the figure can be interpreted as a change in functional connectivity from an overall baseline level. We found that functional connectivity between V5/MT+ and EV-coding regions changed during the course of a trial. When the visual lotteries were shown, functional connectivity increased as evidenced by the positive mean beta estimates. The functional coupling then decreased during the fixation period and became significantly negative at the time of choice response, suggesting a decrease in functional connectivity from the baseline level. Together, these results suggest that changes in functional coupling between these regions can take place during the course of a single trial and that the pattern of changes was modulated by the distinct phases within a trial.

## Discussion

Many choices we make are based on uncertain visual judgments and errors in visual judgment are potentially costly. For instance, we strain to estimate whether anything is in our path while driving under foggy conditions. Since information about the probabilities of occurrence associated with different potential outcomes is not explicitly given, the decision maker has to estimate the probabilities determined by his or her own visual performance, a second-order (Barthelmé and Mamassian, 2009) or metacognitive judgment (Fleming et al., 2012b).

In this study, we investigated a simple version of this form of decision-making. Similar to previous laboratory experiments, subjects were asked to choose between two monetary lotteries on each trial. What is unique is that information about the reward probability associated with each lottery under consideration was not explicitly given in numerical or graphical form. Nor was it learned through sampling. Instead, receipt of reward depended on success in making a judgment about noisy visual information. This requires the subjects to estimate the probability of success given noisy information and to integrate it with rewards that come with it in order to make good decisions. To distinguish this type of lotteries from the more traditional form of economic lotteries, we referred to this kind of lottery as a visual lottery.

### Neural correlates of valuation in the visual lottery decision task

We examined two hypotheses for the valuation of visual lotteries, the common-network hypothesis and the second-order representation hypothesis.

Under the common-network hypothesis, it is assumed that no matter how reward probability information is obtained, whether it is explicitly given in numeric or graphical form, learned from sampling experience, or—as in our experiment—has to be estimated by subjects based on his or her own visual performance, it is represented in a common valuation network that includes the medial prefrontal cortex and the ventral striatum (Bartra et al., 2013; Clithero and Rangel, 2013). Furthermore, probability is integrated with value information so that the network also represents information about EV.

Evidence from the perceptual decision-making literature and metacognition led us to the second-order representation hypothesis, which assumes that the posterior parietal cortex (PPC) and the lateral prefrontal cortex (LPFC) are involved in the metacognitive (second-order) judgment that was required in our visual lottery decisions task (Gold and Shadlen, 2007; Fleming et al., 2012b). Convergent evidence suggests that these regions represent the accumulation and/or strength of the sensory evidence in favor of one direction of motion over the other in visual decision-making tasks (Roitman and Shadlen, 2002; Huk and Shadlen, 2005; Heekeren et al., 2006; Kahnt et al., 2011; Liu and Pleskac, 2011; Filimon et al., 2013). This network has also been shown to represent metacognitive judgments in perceptual and memory tasks (Fleming et al., 2012a; Baird et al., 2013).

The common-network hypothesis and the second-order representation hypothesis provide predictions about the network involved in valuation of the visual lotteries. The two hypotheses are not mutually exclusive. If the common-network hypothesis is supported, this would strengthen the argument that information about probability and its integration with reward value take place in the common valuation network independent of the decision tasks subjects have to perform: probability enters into the valuation network without regard to its source. If the second-order representation hypothesis is confirmed, then the metacognitive estimates of probability are computed in the same networks as in the much studied first-order judgments of direction of motion in the RDM task.

We found that both hypotheses were supported by the data as regions in the lateral prefrontal, dorsomedial prefrontal, and posterior parietal cortices represented both reward probability and reward value of the visual lottery. Further, we found that activation in the dorsomedial prefrontal cortex (dmPFC) and the ventral striatum (vStr)—part of the common valuation network, and the intraparietal sulcus (IPS) and the ventrolateral prefrontal cortex (vlPFC)—part of a network involved in second-order representation, reflected the EV of the visual lotteries when they were presented during a trial. In separate ROI analyses, we also found that the EV representations in these regions were robust under different sensory attributes (motion direction), regardless of the choices subjects made, and the order of lottery presentation. One may argue that we cannot dissociate representations of reward probability from motion coherence level or confidence in making a correct judgment (Kiani and Shadlen, 2009) because both should positively correlate with the probability of making a correct visual judgment. We acknowledge that this is entirely possible. However, both the coherence level and confidence should in principle not be affected by the reward value associated with making a correct judgment. Therefore, the neural representations of EV—the integration of reward probability and reward value—cannot be simply attributed to either the coherence level or confidence alone.

The probability and EV-representations in the common valuation network—the dmPFC and vStr—found in our study were consistent with previous studies where probability of reward was explicitly stated to the subjects or learned from sampling experience (Volz et al., 2003; Knutson et al., 2005; Preuschoff et al., 2006; Berns et al., 2008; Hsu et al., 2009; FitzGerald et al., 2010; Wu et al., 2011). It is worth mentioning that while most value-based decision studies found ventromedial prefrontal cortex (vmPFC) instead of dmPFC, meta-analysis results did also find support for the involvement of dmPFC (Bartra et al., 2013). The dmPFC shown here is in close proximity to the dorsal anterior cingulate cortex (dACC), which was also shown to be involved in valuation (Kennerley et al., 2011; Kolling et al., 2012).

The probability and EV representations found in the second-order representation network—IPS and vlPFC—provide new insight into the role of these regions in valuation during decision-making under uncertainty. Most studies to date found that value representations in these regions are associated with motor actions (e.g., the value associated with making a left or right saccade; Gold and Shadlen, 2007; Kable and Glimcher, 2009). It is not clear whether they also represent the value associated with goods in addition to the action-value representations (Padoa-Schioppa, 2011; but see Tobler et al., 2007; Kahnt et al., 2014 for stimulus-value representations in non-choice tasks). Since information about the motor actions required to indicate subject's choice was not given to the subjects when the lotteries were presented (see Section Materials and Methods), the robust probability and EV representations associated with the visual lotteries under consideration were independent of the specific motor actions required to indicate subjects' decisions. This demonstrates goods-value representations in this network, in addition to action-value representations reported in previous studies. It also points out the possibility that goods-value representations in IPS and vlPFC are task dependent in the sense that the recruitment of this network in goods-value computation depends on whether decisions requires metacognitive judgment of visual performance.

### Dynamic interactions between valuation networks and V5/MT+

Another critical finding in this study is the nature of the functional coupling between area V5/MT+ and the valuation networks. We found a systematic change in functional connectivity between V5/MT+, which had been shown to specialize in visual motion processing (e.g., Tootell et al., 1995), and the EV-coding regions during the course of a trial. Specifically, the functional coupling between these regions increased when the lotteries were presented (presence of visual motion signals) and decreased during the fixation period and at the time of button-press response to indicate choice. Such single-trial dynamics of functional connectivity possibly reflects differences in the demands for visual-motion analysis between different phases of a trial. Together with the EV-coding results, this indicated that the strength of functional coupling between area V5/MT+ and the EV-coding networks is critical to the estimation of performance-based probability and its integration with reward value in the visual lottery decision task. Interestingly, recent results from resting-state fMRI also began to explore the dynamics of functional connectivity between networks and their functional implications to behavioral shifts and adaptive processes (Allen et al., 2014). Future studies may build on these findings to investigate, for example, whether the observed changes in functional coupling can be attributed to the necessity of using visual motion signals to estimate the probability of success and hence reward probability associated the visual lotteries under consideration. If different directions of visual motion are arbitrarily assigned to different reward probabilities, would the pattern of connectivity dynamics be the same as found in this study? Understanding dynamics of functional connectivity between these regions and testing how the pattern of dynamics might change given different task requirements will be important to address the mechanisms for probability computations based on visual motion information.

## Funding

This work was supported by grants from the Ministry of Science and Technology in Taiwan (MOST 99-2410-H-010-013-MY2, MOST 101-2628-H-010-001-MY4) to SW, National Institutes of Health Grants DA027764 to MD, and NEI01889 to LM.

### Conflict of interest statement

The authors declare that the research was conducted in the absence of any commercial or financial relationships that could be construed as a potential conflict of interest.
